# Supplementary material for: Relationship between the Cycle Threshold Value (Ct) of a Salmonella spp. qPCR Performed on Feces and Clinical Signs and Outcome in Horses
Source: Microorganisms. 2023 Jul 30;11(8):1950. doi: 10.3390/microorganisms11081950 (PMC10459194; doi:10.3390/microorganisms11081950)
Supplement: Supplementary file 1 [file microorganisms-11-01950-s001.zip › microorganisms-2301039-supplementary.pdf]

Supplementary Figure S1. Correlation between the qPCR Ct value and the bacterial concentration (log CFU/ml), as determined for three *Salmonella* strains isolated from horse feces samples

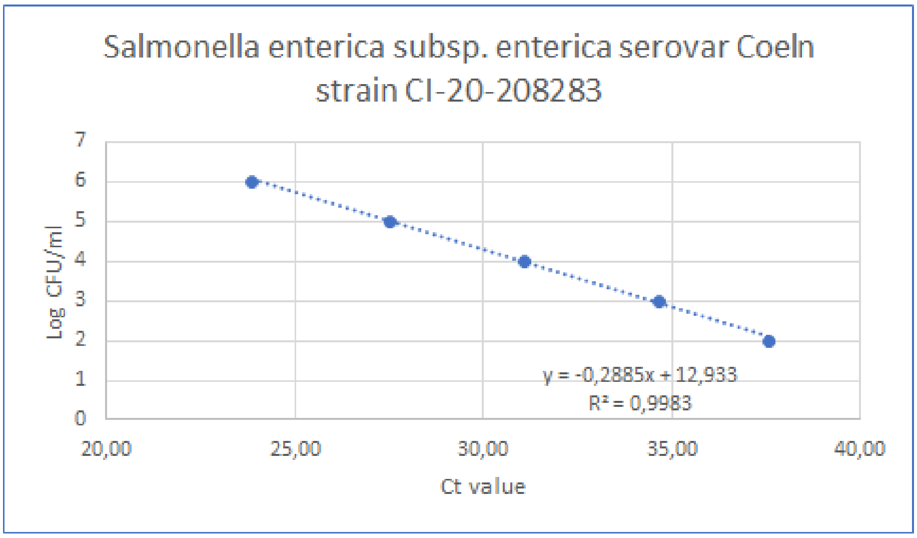

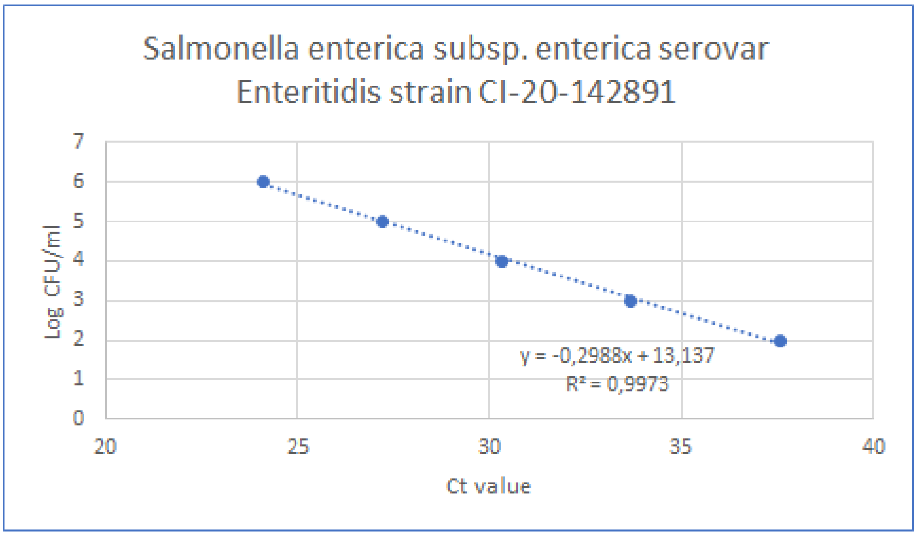

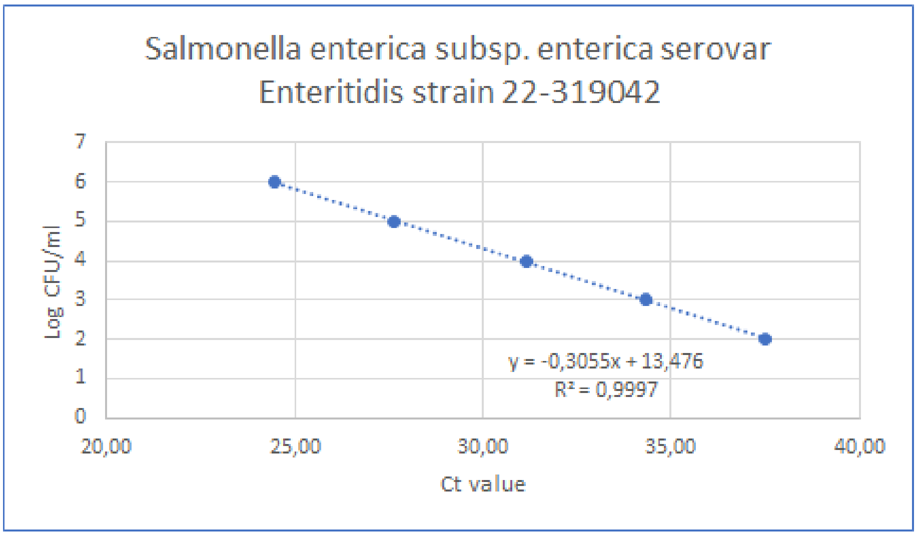

Supplementary Table S1. Comparison of the equation correlating the qPCR Ct value with the bacterial concentration (log CFU/ml), as determined for three *Salmonella* strains isolated from horse feces samples.

Bacterial strain	Log CFU/ml for a Ct value (x) of 30	CFU/ml for x=30	% mean	Slope	Efficiency
S. enterica Coeln CI-20-208283	4.28	18967.06	104.7%	-0.29	94.5%
S. enterica Enteritidis CI-20-142891	4.17	14893.61	82.2%	-0.30	99.3%
S. enterica Enteritidis 22-319042	4.31	20464.45	113.0%	-0.31	102.1%
**Mean**	**4.25**	**18108.37**	**100.0%**		
**SD**	**0.07**	**2882.98**	**15.9%**		
